# Maturity Grading and Identification of *Camellia oleifera* Fruit Based on Unsupervised Image Clustering

**DOI:** 10.3390/foods11233800

**Published:** 2022-11-25

**Authors:** Xueyan Zhu, Deyu Shen, Ruipeng Wang, Yili Zheng, Shuchai Su, Fengjun Chen

**Affiliations:** 1 School of Technology, Beijing Forestry University, Beijing 100083, China; 2 Beijing Laboratory of Urban and Rural Ecological Environment, Beijing Forestry University, Beijing 100083, China; 3 Key Laboratory of Silviculture and Conversation, Ministry of Education, Beijing Forestry University, Beijing 100083, China

**Keywords:** *Camellia oleifera*, maturity grading, maturity identification, unsupervised image clustering, harvesting process

## Abstract

Maturity grading and identification of *Camellia oleifera* are prerequisites to determining proper harvest maturity windows and safeguarding the yield and quality of Camellia oil. One problem in *Camellia oleifera* production and research is the worldwide confusion regarding the grading and identification of *Camellia oleifera* fruit maturity. To solve this problem, a *Camellia oleifera* fruit maturity grading and identification model based on the unsupervised image clustering model DeepCluster has been developed in the current study. The proposed model includes the following two branches: a maturity grading branch and a maturity identification branch. The proposed model jointly learns the parameters of the maturity grading branch and maturity identification branch and used the maturity clustering assigned from the maturity grading branch as pseudo-labels to update the parameters of the maturity identification branch. The maturity grading experiment was conducted using a training set consisting of 160 *Camellia oleifera* fruit samples and 2628 *Camellia oleifera* fruit digital images collected using a smartphone. The proposed model for grading *Camellia oleifera* fruit samples and images in training set into the following three maturity levels: unripe (47 samples and 883 images), ripe (62 samples and 1005 images), and overripe (51 samples and 740 images). Results suggest that there was a significant difference among the maturity stages graded by the proposed method with respect to seed oil content, seed soluble protein content, seed soluble sugar content, seed starch content, dry seed weight, and moisture content. The maturity identification experiment was conducted using a testing set consisting of 160 *Camellia oleifera* fruit digital images (50 unripe, 60 ripe, and 50 overripe) collected using a smartphone. According to the results, the overall accuracy of maturity identification for *Camellia oleifera* fruit was 91.25%. Moreover, a Gradient-weighted Class Activation Mapping (Grad-CAM) visualization analysis reveals that the peel regions, crack regions, and seed regions were the critical regions for *Camellia oleifera* fruit maturity identification. Our results corroborate a maturity grading and identification application of unsupervised image clustering techniques and are supported by additional physical and quality properties of maturity. The current findings may facilitate the harvesting process of *Camellia oleifera* fruits, which is especially critical for the improvement of Camellia oil production and quality.

## 1. Introduction

As an essential raw material source of edible oil, *Camellia oleifera* is of momentous edible woody oil seed crop in China, India, Japan, and Korea [1,2]. Several studies have reported Camellia oil can not only be used as cooking oil but can also be used as a drug carrier and healthcare product [3,4]. Camellia oil has a high content of unsaturated fatty acids (e.g., oleic acid and omega-6-linoleic acid) as well as natural bioactive components (e.g., squalene, polyphenols, and sasanquasaponin) that have promoting effects on health care and anti-aging [5,6,7,8]. Therefore, the demand for high-quality Camellia oil has been continuously increasing in recent years [9]. The productivity and quality of Camellia oil are not only solely related to factors such as variety, climate, and squeezing technology but also inextricably related to maturity [10]. As the fruit matures, the oil content of *Camellia oleifera* fruits was increased first and then decreased [11]. However, the acidity increases during the ripening of *Camellia oleifera*, reducing the quality of the produced Camellia oil [12]. Therefore, the yield and quality of Camellia oil are determined by the maturity stage of the harvested fruit. As a rule of thumb, both early and later-harvested *Camellia oleifera* fruits exhibit decreased oil content and quality [13,14]. Hence, accurate identification of *Camellia oleifera* fruit maturity has to be made.

Based on the accumulation of long-term practical experience, the traditional Chinese solar terms (e.g., Autumn Equinox, Cold Dews, Frost’s Descents, and Winter Begins) were commonly used to determine the maturity of different *Camellia oleifera* cultivars [15]. For instance, the traditional Chinese solar terms Frost’s Descents has been considered to be a mature symbol of numerous *Camellia oleifera* cultivars, such as Huaxin, Huajin, Xianglin 1, Xianglin 27, Xianglin 97, and Xianglin 210. Moreover, the traditional Chinese solar term Cold Dews has been used to determine the maturity of different *Camellia oleifera* cultivars, including Xianglin 104, Xianglin 106, Xianglin 107, and Xianglin 131. However, extensive research has found that with global climate change, the phenology of *Camellia oleifera* has altered gradually, which will result in the currently recommended maturity based on traditional Chinese solar terms is not applicable [16,17]. Accordingly, a stable *Camellia oleifera* fruit maturity identification method without being affected by climate change is urgently needed.

For a long time, the oil content has been considered one of the most critical parameters to determine the quality of *Camellia oleifera*, which can be applied as a stable and climate-independent parameter to determine the *Camellia oleifera* fruit maturity stages [18,19,20]. Based on literature studies, *Camellia oleifera* has an oil content that will significantly increase after entering maturity, and then it will slowly decrease due to being overripe [21]. Conventionally, oil content measurement of *Camellia oleifera* fruit samples is carried out in the laboratory using chemical experiments [22,23]. Despite its stability and efficacy, maturity determine based on oil content suffers from the following several major drawbacks: labor-intensive, time-consuming, sample damage, prone to human error, and not environmentally sustainable.

Based on the facts above, several researchers have attempted to produce a technological breakthrough that can non-invasive identify the maturity of oil fruits such as oil palm [24,25,26] and olive [27,28]. Tugnolo et al. [29] used visible/near-infrared imaging in a study that automatic identifying the maturity of olive fruits. The sensitivity, specificity, and accuracy of identifying the maturity of olive fruits for the method were 86%, 87%, and 87%, respectively. Raj et al. [30] proposed a method that identification the maturity of oil palm fruitlets based on carotene content from Raman spectra. They reported that the overall accuracy of identification for the best model for oil palm maturity identification was 100%. Jiang et al. [31] conducted a study in which they used a hyperspectral imaging system to access the maturity of *Camellia oleifera* fruit. The *Camellia oleifera* fruit maturity identification results indicated that their model was 81.2% accurate. Although the non-invasive maturity identification techniques used in this research works well, the techniques usually require expensive specialized equipment. However, it is very difficult for the grower to obtain this specialized equipment.

To date, in various fruit maturity grading and identification systems, deep learning-based computer vision has become a substitute for subjective experience, destructive testing, and non-invasive testing, being objective, consistent, efficient, and economical [32,33]. Deep learning-based computer vision has also been used for the identification of the maturity of different oil fruits, including oil palm [34,35,36], coconut [37], and olive [38]. Khosravi et al. [39] designed a deep convolutional neural network (CNN) in a study that identified the maturity of on-branch olives for Zard and Roghani cultivars. The results of their study showed that the overall accuracy and prediction time for the model were 91.91% and 12.64 ms, respectively. Parvathi et al. [40] described a method based on Faster R-CNN for the detection of two important maturity stages for coconuts in complex backgrounds. In another study, Mubin et al. [41] described a method based on deep learning for the objective and accurate detection of the maturity of oil palms. The results indicated that the accuracy of detection for young and mature oil palms was 95.11% and 92.96%, respectively.

Based on the above-mentioned, it was noted that the majority of published papers target applications of deep learning techniques in the maturity detection of oil fruits, which have maturity grading standards. Up to now, far too little attention has been paid to deep learning for the maturity identification of *Camellia oleifera* fruits that has no maturity grading standard. The review of the previous studies has failed to grade and identification the maturity of *Camellia oleifera* fruits based on images. Therefore, the objective of the present study is to demonstrate a *Camellia oleifera* fruit maturity grading and identification method based on unsupervised image clustering. The contributions of this paper are as follows:

(1) Using an unsupervised image clustering method to grade and identification the maturity of *Camellia oleifera* fruits;
(2) Conjoint analysis was performed for the physical and quality properties of *Camellia oleifera* fruits with different maturity;
(3) The Gradient-weighted Class Activation Mapping (Grad-CAM) visualization analysis reveals the critical regions for *Camellia oleifera* fruit maturity identification.

## 2. Materials and Methods

### 2.1. Fruit Sample Preparation

**Figure 1:**
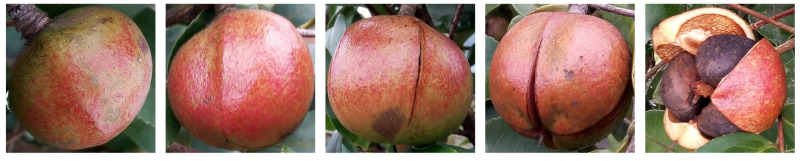
Images example of *Camellia oleifera* fruit.

The data used in this study was a set of fruit images and samples of the Huaxin variety obtained from the state-owned *Camellia oleifera* Forestry Farm of Huang Lawn (111°36′8″ N, 27°6′58″ E), Shaoyang City, Hunan Province, China. Color images and samples data of the *Camellia oleifera* fruit were collected 16 times (once every 5 days) starting from early August to mid-November 2020. In image acquisition, the capturing distance between the digital camera built-in smartphone (Xiaomi 10) and the *Camellia oleifera* fruit was approximately 10–30 cm, and the collected images were saved in a 24-bit true-color JPEG format. The images were collected under the condition of sufficient light. Moreover, the digital camera focus mode, white balance, and sensitivity were set to auto, the shutter time was set to 1/2000 s and the exposure compensation was set to 0. Additionally, 20 defect-free fruit samples of *Camellia oleifera* with similar sizes were randomly selected, imaged, and picked during each data collection. After picking fruit samples, physical and quality properties were evaluated. In this study, 320 *Camellia oleifera* fruit samples (16 groups) with different maturity stages were selected. A total of 2788 *Camellia oleifera* fruit images covering the whole mature period were collected, of which 320 images (one image per sample) were corresponding to the sample fruits. The other 2468 *Camellia oleifera* fruit images were randomly collected images of *Camellia oleifera* fruits with phenotypic characteristics roughly similar to the fruit samples. Moreover, the 2468 images were taken from the same *Camellia oleifera* trees of the same variety in the same experimental area as the 320 *Camellia oleifera* fruit sample images mentioned above. The image acquisition equipment, acquisition parameters, and other experimental conditions were identical to the acquisition condition of 320 *Camellia oleifera* fruit sample images. Examples of *Camellia oleifera* images are presented in Figure 1.

### 2.2. Dataset Details

The *Camellia oleifera* fruit images and samples were divided into the following two sets: a training set (TN) and a testing set (TS), as presented in Table 1. The training set consists of 160 *Camellia oleifera* fruit samples (obtained by randomly selecting 10 samples from each group) and 2628 *Camellia oleifera* fruit images. Among the 2628 *Camellia oleifera* fruit images, there were 160 *Camellia oleifera* fruit images that were corresponding to each of the 160 *Camellia oleifera* fruit samples in the training set. A total of 160 *Camellia oleifera* fruit images and samples were included in the testing set. The *Camellia oleifera* fruits in the testing set were divided into the unripe testing set (Unripe_TS), the ripe testing set (Ripe_TS), and the overripe testing set (Overripe_TS) according to seed oil content [42]. Moreover, the *Camellia oleifera* fruit samples and images of the training set were not manually divided into different maturity according to seed oil content. This is because the unsupervised image clustering technique was used in this study to grade and identified the maturity of *Camellia oleifera* fruits. The typical characteristic of unsupervised image clustering is that the training set is without manual intervention.

### 2.3. Proposed Method of Fruit Maturity Grading and Identification

**Figure 2:**
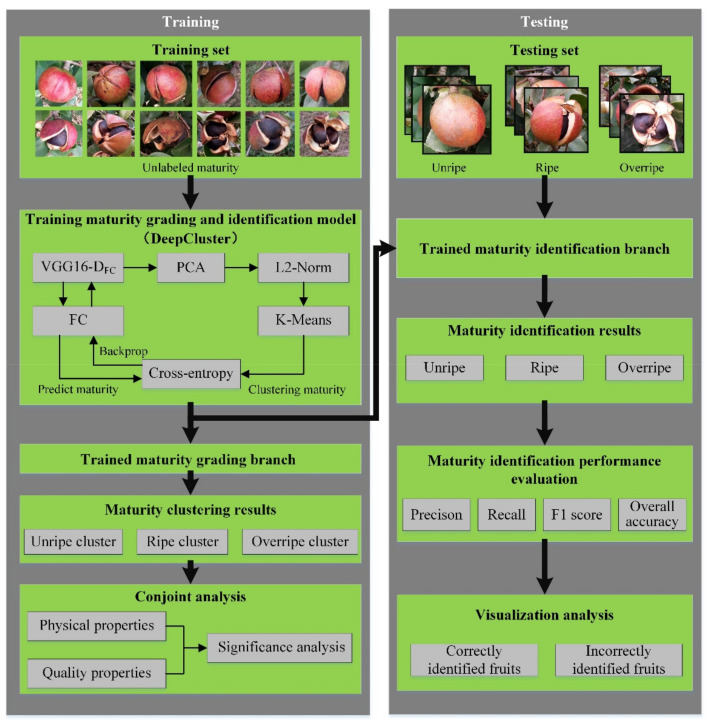
Overview of all processes in the proposed method of *Camellia oleifera* fruit maturity grading and identification.

The proposed method aims to propose a *Camellia oleifera* fruit maturity grading and identification method based on unsupervised learning. It was divided into two stages, namely, maturity grading and identification, and conjoint analysis of physical and quality properties, where the input for maturity grading was acquired from the *Camellia oleifera* fruit images, and the input for physical and quality property conjoint analysis were acquired from the *Camellia oleifera* fruit samples. In the maturity grading stage, the DeepCluster method was applied to grading *Camellia oleifera* fruit samples and images in training set into different maturity. In the conjoint analysis of the physical and quality properties stage, the ANOVA coupled with Duncan’s test was applied to analyze the differences in physical and quality properties of *Camellia oleifera* fruit with different maturity stages clustered by DeepCluster. An overview of all processes in the proposed method of *Camellia oleifera* fruit maturity grading and identification is depicted in Figure 2.

#### 2.3.1. Grading and Identification of Fruit Maturity

**Figure 3:**
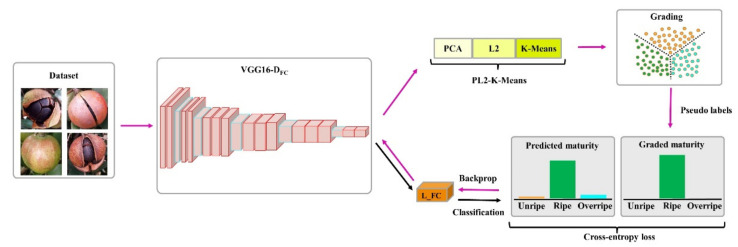
Overview of all processes in the proposed *Camellia oleifera* fruit maturity grading and identification model based on unsupervised image clustering, VGG16-D_FC_ model indicates the VGG16 network with the last fully connected layer removed, L_FC denotes the last fully connected layer of VGG16, L2 represents the L2 normalization.

*Camellia oleifera* fruit maturity was graded and identified by DeepCluster [43], an end-to-end unsupervised image clustering model that jointly learns the parameters of neural network (VGG16-D_FC_) and the cluster assignments (K-Means) of the resulting features. As shown in Figure 3, the DeepCluster iteratively groups the features with the K-Means clustering algorithm and updates the parameters of the network using the subsequent assignments as supervision. The DeepCluster model mainly includes the following two main branches: the maturity grading branch (VGG16-D_FC_ and PL2-K-Means) and the maturity identification branch (VGG16-D_FC_ and L_FC). The maturity grading branch clustering the maturity of *Camellia oleifera* fruits into different stages based on the features extracted by VGG16-D_FC_. Moreover, the maturity identification branch identifies the maturity of *Camellia oleifera* fruits based on the features extracted by VGG16-D_FC_.

As shown in Figure 3, the DeepCluster model receives *Camellia oleifera* fruit images with unlabeled maturity stages as input data. In the feature extraction stages, the features of *Camellia oleifera* fruit images in the dataset are extracted. Feature extraction was carried out based on the features extracted by the VGG16-D_FC_ model because these features have been used successfully in previous works. The initial weights of the VGG16-D_FC_ model were generated by random initialization. Because previous studies have shown that these random features perform much better on standard transfer tasks than on random levels [44]. After the feature extraction, the extracted features (4096 dimensional) of *Camellia oleifera* fruit images are output through the penultimate fully connected layer of the VGG16-D_FC_ model [45].

In the feature selection stages, the features extracted were selected. A total of 4096 features were extracted in the feature extraction stage. This leads to complicated clustering processes that also require time-consuming computations. Therefore, feature selection was needed to obtain the critical features with an important contribution to clustering. In this study, the feature selection model—principal component analysis (PCA)—was performed to significantly reduce the number of features. Moreover, the PCA method has been widely applied in many objects related to this work [46,47,48]. Afterward, normalization was implemented using L2 normalization to minimize the effect of singular features [49]. Because L2 normalization has been successfully used in previous works related to normalization [50,51].

The normalized features were generated by applying the L2 normalization and subsequently used as input in the feature clustering stage. In this study, the K-Means clustering algorithm was implemented to cluster the normalized features into *k* distinct groups based on a geometric criterion. Moreover, the K-Means clustering algorithm has been successfully applied in studies related to this work [52,53,54]. After clustering, each *Camellia oleifera* image *i* in the dataset has a uniquely determined clustering assignment *y_i_*. The optimal cluster assignments (y_i_*)*_n_*_≤*N*_ and *d* × *k* centroid matrix C* can be jointly learned using the objective function in Equation (1).

(1) $$\underset{C\in {\mathbb{R}}^{d\times k}}{\min}\frac{1}{N}\sum_{i=1}^{N}\underset{y_{i}\in {\left\{0,1\right\}}^{k}}{\min}{\left\|f_{\theta }(x_{i})-Cy_{i}\right\|}_{2}^{2}$$

where $y_{n}^{T}1_{k}=1$ and *N* is the total number of *Camellia oleifera* images in the dataset, *x_i_* denotes the *i*th *Camellia oleifera* image in the dataset, *k* denotes the clustering number, *C* denotes the *d* × *k* centroid matrix, $f_{\theta }$ denotes the convnet mapping, θ is the set of corresponding parameters of convnet mapping, *y_i_* is the clustering assignment of *i*th *Camellia oleifera* fruit image.

Then, these assignments were used as pseudo-labels to jointly learn the classifier parameters *W* and the mapping parameters θ using the objective function in Equation (2). Moreover, this objective function is optimized by mini-batch stochastic gradient descent and backpropagation to calculate the gradient.

(2) $$\underset{\theta ,W}{\min}\frac{1}{N}\sum_{i=1}^{N}l(g_{W}(f_{\theta }(x_{i})),y_{i})$$

where *l* denotes the negative log-softmax function, and *g_w_* denotes the parametrized classifier.

#### 2.3.2. Determination of Physical Properties

After *Camellia oleifera* fruit sample picking, the physical properties of each fruit sample were determined individually. Physical properties were transverse diameter, vertical diameter, dry seed weight, and moisture content of *Camellia oleifera* fruit samples. The transverse and vertical diameters of each *Camellia oleifera* fruit sample were determined on a digital caliper (Deguqmnt, MNT-150T, Hanover, Germany). To acquire the dry seeds, the fresh seeds were deactivated at 105 °C for 15 min and baked at 70 °C for 72 h [13]. Similarly, the fresh seed weight (*W_f_*) and dry seed weight (*W_d_*) of each *Camellia oleifera* fruit sample were determined with an electronic balance (Shimadzu, UW2200H, Kyoto, Japan). Next, the moisture content (*M_c_*) was determined using the following equation:

(3) $$M_{c}=\frac{W_{f}-W_{d}}{W_{f}}\times 100\%$$

#### 2.3.3. Determination of Quality Properties

Once *Camellia oleifera* fruit samples were taken to the laboratory, the quality property included seed oil content, seed soluble protein content, seed soluble sugar content, and seed starch content were measured for 10 samples in each group. Because the quality properties of individual *Camellia oleifera* fruit samples were difficult to be accurately measured. The oil content in *Camellia oleifera* seed was determined following exhaustive chemical extraction using a Soxtec 2050 extraction system (Foss Analytical, Hillerød, Denmark), as described by Yang et al. [7]. Next, the soluble protein content of the *Camellia oleifera* seed was determined by Coomassie brilliant-blue G-250 staining method on a UV-vis spectrophotometer (Agilent Technologies, Santa Clara, CA, USA) [55]. Subsequently, the soluble sugar content and starch content of the *Camellia oleifera* seed were determined by the anthrone colorimetric method on a UV-vis spectrophotometer (Agilent Technologies, Santa Clara, CA, USA), as described in detail by Zhang et al. [13]. Moreover, the quality properties of each sample in the group were represented approximately by the quality properties within the group.

#### 2.3.4. Conjoint Analysis of Maturity with Physical and Quality Properties

In the fruit maturity clustering stage, the *Camellia oleifera* fruit images are clustered into different maturity stages based on the phenotypic properties extracted by the neural network. Different maturity stages of *Camellia oleifera* fruits had differences in image properties, but it remains to be verified whether differences existed in physical and quality properties. Therefore, analysis of variance (ANOVA) was performed using SPSS software (IBM SPSS 20, Chicago, IL, USA) for the physical and quality properties of *Camellia oleifera* fruit samples in different maturity. Duncan’s multiple comparison tests were used to compare the significance (*p* ≤ 0.05) in physical and quality properties among different clusters of *Camellia oleifera* fruits.

### 2.4. Maturity Identification Performance Evaluation Index

To comprehensively evaluate the effectiveness of the proposed method, four evaluation indexes of image identification, including the precision (*Prec*), recall (*Rec*), F1 score (*F1_score_*), and overall accuracy (*OA_cc_*), were used. According to [56], the *Prec*, *Rec*, *F1_score_*, and *OA_cc_* can be calculated through the following equations:

(4) $$\mathrm{Prec}=\frac{\mathrm{TP}}{\mathrm{TP}+\mathrm{FP}}\times 100\%$$

(5) $$\mathrm{Rec}=\frac{\mathrm{TP}}{\mathrm{TP}+\mathrm{FN}}\times 100\%$$

(6) $${F1}_{\mathrm{score}}=2\times \frac{\mathrm{Prec}\times \mathrm{Rec}}{\mathrm{Prec}+\mathrm{Rec}}\times 100\%$$

(7) $${\mathrm{OA}}_{\mathrm{cc}}=\frac{\mathrm{TP}+\mathrm{TN}}{\mathrm{TP}+\mathrm{FP}+\mathrm{FN}+\mathrm{TN}}\times 100\%$$

where *TP* represents the positive samples that are correctly identified as a positive class, *FP* denotes the negative samples predicated as a positive class by the proposed method, *FN* means the positive samples predicted as a negative class, and *TN* denotes the negative samples predicted as a negative class by the proposed method.

### 2.5. Model Visualization

The fruit maturity grading and identification model extract extensive information from the *Camellia oleifera* fruit images, and these feature data can be visualized and interpreted by Grad-CAM. This method has been successfully applied in the image analysis of lychee and *Camellia oleifera* cultivar identification [57,58]. The Grad-CAM algorithm obtains a location map that can interpret the key identification regions in the image by weighting and processing the activation feature maps of the last layer according to their weights [59]. The Grad-CAM visualization explains that regions of the image the fruit maturity grading and identification model focused on when determining the *Camellia oleifera* fruit to be a specific maturity.

## 3. Results and Discussion

### 3.1. Implementation Details

The *Camellia oleifera* fruit maturity grading and identification model was implemented on the Ubuntu 16.04 system and the following hardware configurations: an NVIDIA GeForce GTX 1080 Ti GPU and the memory of 11 G. To construct the *Camellia oleifera* fruit maturity grading and identification model, we used the Pytorch 1.5.1 platform, which is based on Python ver. 3.6.13. During the model training process, the learning rate was set to 0.1 and the momentum to 0.9.

### 3.2. Fruit Maturity Grading Results

**Figure 4:**
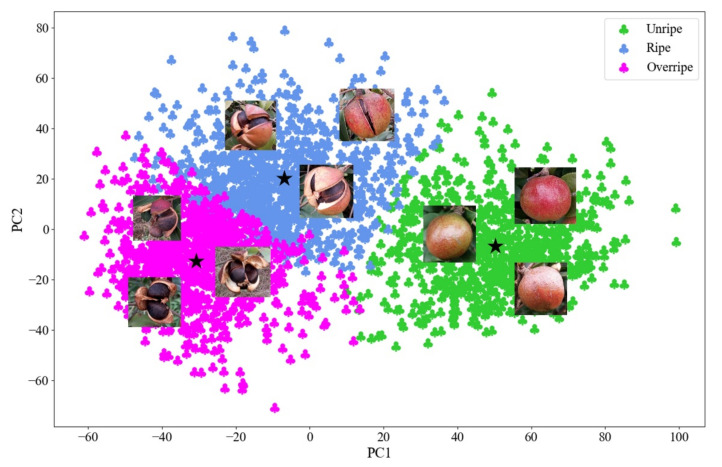
The maturity grading results of the training set. Here, the black pentagram represents the center of each maturity stage, and the *Camellia oleifera* images represent the fruit samples of each maturity stage.

In the model training stages, the 2628 *Camellia oleifera* fruit images in the training set were used as input to the DeepCluster. After training, the 2628 *Camellia oleifera* fruit images in the training set were automatically graded into unripe (883 images), ripe (1005 images), and overripe (740 images), as shown in Figure 4. Then, from 2628 *Camellia oleifera* fruit images after clustering, we located the images that corresponded to each of the 160 *Camellia oleifera* fruit samples in the training set. The 160 *Camellia oleifera* fruit samples in the training set were graded into unripe, ripe, and overripe with the numbers 47, 62, and 51. As shown in Figure 4, the features of the unripe *Camellia oleifera* are mainly distributed in the right half of the cluster image, and the features of the ripe *Camellia oleifera* are mainly distributed in the middle of the cluster image. Meanwhile, the features of overripe *Camellia oleifera* are mainly distributed in the left half of the cluster image.

By observing the maturity grading results along with fruit samples of each maturity stage in Figure 4, we can find that the phenotypic properties of *Camellia oleifera* fruit with the same maturity were approximately similar, while differences existed in the phenotypic properties of *Camellia oleifera* fruit of different maturity. It can be seen that feature extraction by VGG16-D_FC_ and feature clustering by K-Means plays an important role in *Camellia oleifera* maturity clustering. Among the three different maturity stages of *Camellia oleifera*, the *Camellia oleifera* fruits that are unripe have a sleek, and indehiscent peel. The *Camellia oleifera* fruits that are ripe have a sleek, and slightly cracked peel, and the *Camellia oleifera* seeds are slightly exposed. The *Camellia oleifera* fruits with overripe have a rough and obviously cracked peel, and the *Camellia oleifera* seeds are obviously exposed. These results indicated that the phenotypic properties of different clusters of *Camellia oleifera* fruits that are significantly different after clustering using the DeepCluster model. It also confirms the effectiveness of using the DeepCluster model to automatically grade the maturity of *Camellia oleifera*.

**Figure 5:**
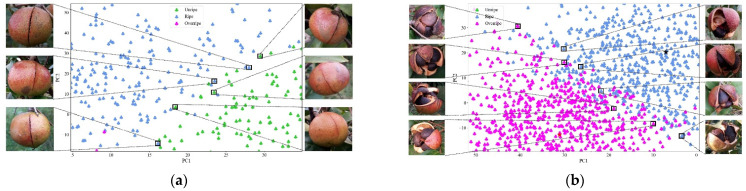
The distribution of *Camellia oleifera* fruits in the overlapping regions and the example images located in overlapping regions: (**a**) the distribution of *Camellia oleifera* fruit images in unripe and ripe overlap regions and the example images located in overlapping regions, and (**b**) the distribution of *Camellia oleifera* fruit images in ripe and overripe overlap regions and the example images located in overlapping regions.

According to Figure 4, there exists overlap regions between unripe and ripe as well as between ripe and overripe. However, between unripe and overripe there hardly exists overlap regions. To further present the details of the overlap regions, several examples of the overlap regions are depicted in Figure 5. Figure 5a shows the distribution of *Camellia oleifera* fruit images in unripe and ripe overlap regions and the example images located in overlapping regions. Figure 5b shows the distribution of *Camellia oleifera* fruit images in ripe and overripe overlap regions and the example images located in overlapping regions. It is evident from Figure 5a that the *Camellia oleifera* of different maturity stages in the overlapping regions (unripe and ripe) have slight cracks. Moreover, as shown in Figure 5b, the *Camellia oleifera* of different maturity stages in the overlapping regions (ripe and overripe) were obviously cracked, and the *Camellia oleifera* seeds were exposed. Therefore, we speculate that when grading the maturity of *Camellia oleifera*, the DeepCluster model not only considered the crack of the fruit, but also the appearance factors such as peel color and texture.

### 3.3. Conjoint Analysis Results

This section analyzes the physical and quality properties of *Camellia oleifera* fruit samples with different maturity stages. Table 2 gives information about the physical properties of *Camellia oleifera* fruit samples within three different maturity stages graded by DeepCluster. As the table illustrates approximately, no significant differences were found in transverse and vertical diameter values with *Camellia oleifera* maturity stages. As other authors have observed, there were no significant differences in the transverse diameter and vertical diameter of the *Camellia oleifera* with different maturity stages [60,61]. For dry seed weight and moisture content, there were significant differences among the maturity stages. This conclusion was in agreement with the previous findings of Jiang et al. [31]. According to the above, the physical properties of *Camellia oleifera* fruit samples with different maturity stages automatically graded by DeepCluster were basically consistent with previous studies [62]. This result demonstrates the validity of the maturity grading method from the perspective of physical properties.

The quality properties of *Camellia oleifera* fruit samples with three different maturity stages graded by DeepCluster are presented in Table 3. As the table gives information about quality properties, there is a remarkable difference between maturity stages and quality properties. The seed oil content is of particular significance for *Camellia oleifera*. As other authors have observed, the seed oil content of *Camellia oleifera* increased significantly and then decreased slightly with the maturation of the fruits [11]. The highest seed oil content was 46.05%, which was obtained from the ripe stage. The seed oil content decreased amounted to 1.63% from the ripe to the overripe stage. This conclusion is consistent with the findings of Jiang et al. [31]. In addition, the seed-soluble protein content gradually increased as the fruit matured, which was consistent with the results of previous studies [11]. Moreover, as the increases of fruit maturity stage, the seed-soluble sugar content and seed starch content gradually decrease [63]. These results indicate the validity of *Camellia oleifera* maturity as graded by DeepCluster in terms of quality properties.

Based on the data in Table 2 and Table 3, and the analysis above, it can be seen that the physical and quality properties of *Camellia oleifera* fruits at different maturity stages are basically consistent with previous findings. This conclusion verifies the effectiveness of the *Camellia oleifera* fruit maturity grading and identification method based on unsupervised learning. At the same time, it can be found that the seed oil content of *Camellia oleifera* fruits was optimal at the ripe stage. Therefore, the highest yield was likely obtained by harvesting *Camellia oleifera* fruits at ripe maturity.

### 3.4. Maturity Identification Performance Evaluation

**Figure 6:**
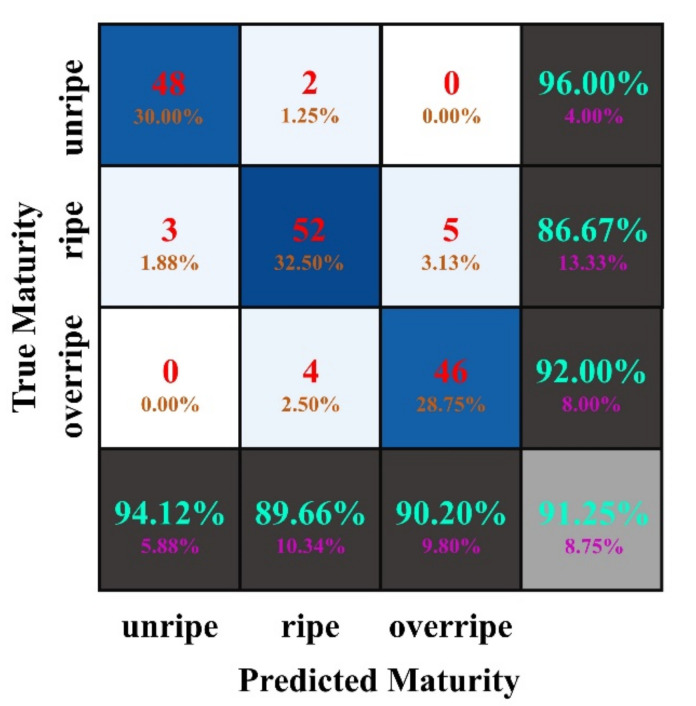
Confusion matrix for identification *Camellia oleifera* fruit maturity in the testing set.

**Figure 7:**
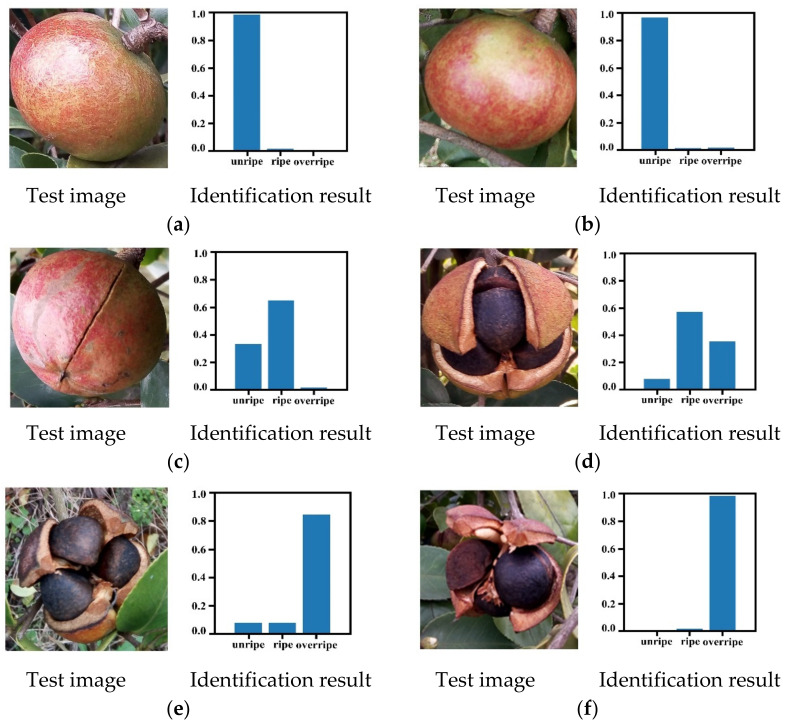
Maturity identification results of some *Camellia oleifera* fruit example images in testing set: (**a**,**b**) denotes maturity identification results of unripe *Camellia oleifera* fruits, (**c**,**d**) denotes maturity identification results of ripe *Camellia oleifera* fruits, (**e**,**f**) denotes maturity identification results of overripe *Camellia oleifera* fruits.

An experiment was designed to quantitatively evaluate the maturity identification ability of the DeepCluster model. In total, 160 sample *Camellia oleifera* fruits (50 unripe, 60 ripe, and 50 overripe) in the testing set were used to perform the evaluation maturity identification. In the identification problem, a confusion matrix is often used as a way of evaluating the performance of the proposed method [64,65]. Thus, the confusion matrix was used in this study to quantity evaluate the maturity identification results of *Camellia oleifera*. Figure 6 indicates the maturity identification results of the model in the form of a confusion matrix, and the maturity identification results of some *Camellia oleifera* fruit example images are presented in Figure 7.

Generally, the lower the sum of the off-diagonal elements in the confusion matrix, the higher the accuracy of identification would be. As shown in the confusion matrix, the DeepCluster model trained with the *Camellia oleifera* images without labeled maturity can properly identify the maturity of *Camellia oleifera*. Of the 160 *Camellia oleifera* fruit samples with different maturity, 146 were correctly identified, while only 14 were mistakenly identified. Moreover, the misidentification mainly occurs between the adjacent maturity stages such as unripe and ripe, ripe, and overripe. For instance, of the 50 *Camellia oleifera* sample images with unripe maturity, 48 were correctly identified and 2 were mistakenly identified as ripe. Figure 7a,b show the maturity identification results of some unripe *Camellia oleifera* fruits in the testing set. For 60 ripe *Camellia oleifera* sample images, the proposed method correctly identified 52 of the *Camellia oleifera* sample images, and the remaining 8 *Camellia oleifera* sample images were identified as unripe (3) and overripe (5). Figure 7c,d show the maturity identification results of some ripe *Camellia oleifera* fruits in the testing set. Moreover, the *Camellia oleifera* fruit examples depicted in Figure 7c,d are the most challenging examples for the model identification of ripe *Camellia oleifera* fruits. Actually, the *Camellia oleifera* fruit shown in Figure 7c has features similar to unripe, while the *Camellia oleifera* fruit in Figure 7d was close to overripe. In addition, of the 50 *Camellia oleifera* sample images with overripe maturity, 46 were correctly identified and 4 were mistakenly identified as ripe. Figure 7e,f show the maturity identification results of some overripe *Camellia oleifera* fruits in the testing set. We conjectured that these misidentifications were caused because the phenotypic properties of adjacent maturity *Camellia oleifera* visually appear more similar to each other.

In this study, the following four evaluation indices were employed to quantitatively evaluate the performance of the proposed *Camellia oleifera* maturity identification method: precision, recall, F1 score, and overall accuracy. Table 4 summarizes the performance evaluation results of *Camellia oleifera* maturity identification by implementing the proposed method. Accordingly, the proposed method could identify *Camellia oleifera* fruit sample images into three maturity stages with an overall accuracy of 91.25%. For each maturity stage identification of *Camellia oleifera*, different results were obtained by the proposed method. For example, in the proposed method, the unripe group obtained the best identification with 94.12% precision, 96.00% recall, and 95.05% F1 score. The overripe group obtained the suboptimal identification and achieved precision, recall, and F1 score of 90.20%, 92.00%, and 91.09%, respectively. However, the ripe group reached the value of precision, recall, and the F1 score, which was lower than both other maturity stages. This was due to the fact that the ripe group of *Camellia oleifera* can be confused with both the unripe and overripe groups of *Camellia oleifera*. Based on the *Camellia oleifera* fruit maturity identification results of Figure 7, Table 4, and the analysis above, it can be seen that the proposed method can be used to effectively identify the maturity of *Camellia oleifera* fruits.

### 3.5. Maturity Identification Visualization Analysis

**Figure 8:**
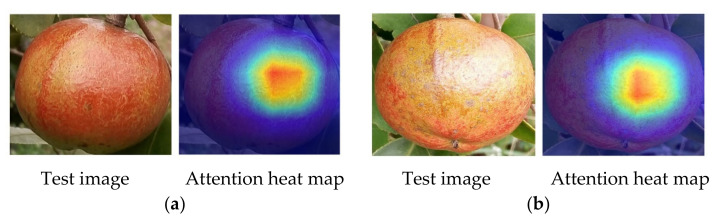

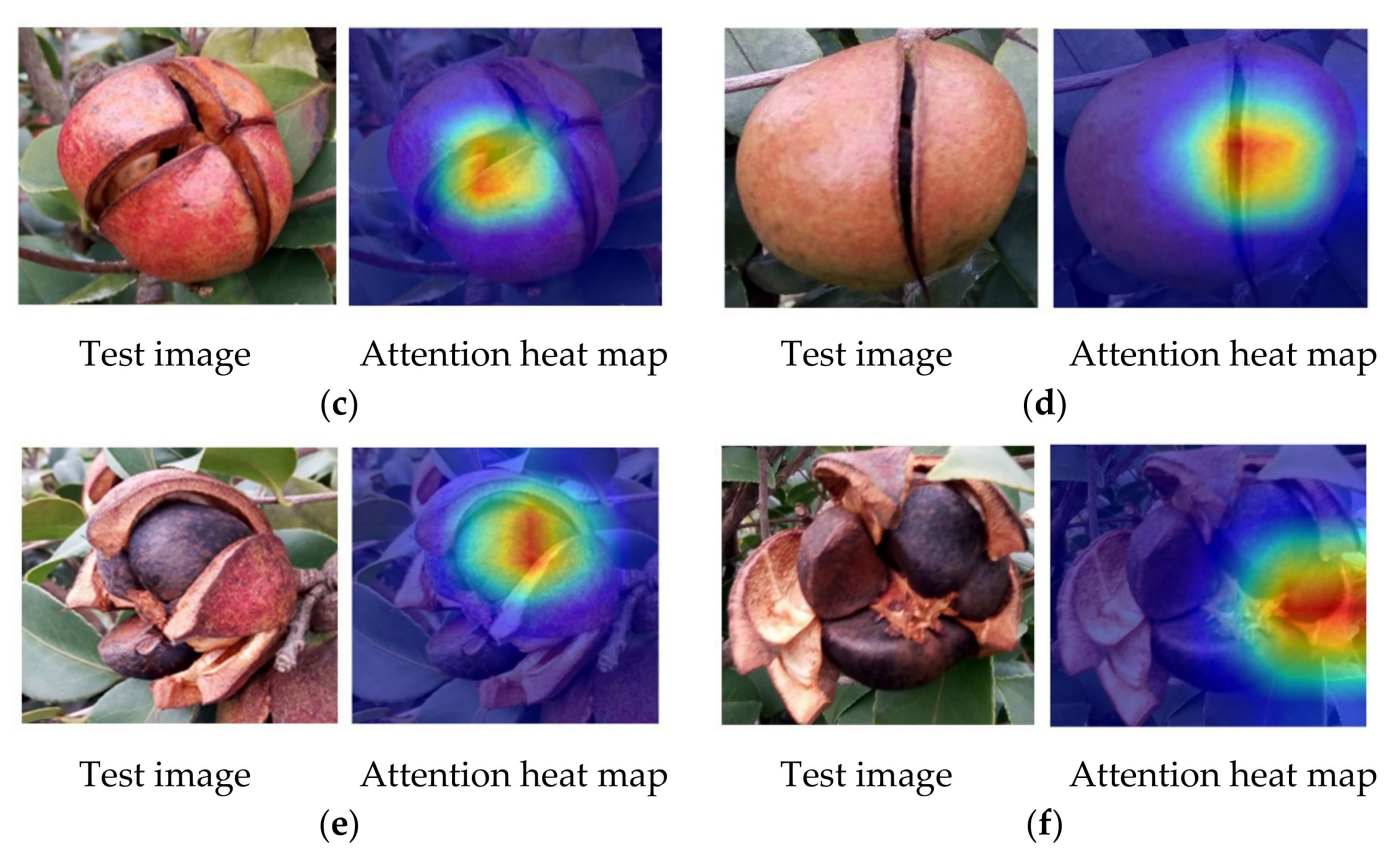
Heat maps of some *Camellia oleifera* fruit example images: (**a**,**b**) denotes example heat maps of unripe *Camellia oleifera* fruits, (**c**,**d**) denotes example heat maps of ripe *Camellia oleifera* fruits, (**e**,**f**) denotes example heat maps of overripe *Camellia oleifera* fruits. Here, warm colors indicate that the region has a strong contribution to the maturity prediction.

According to the heat maps of some *Camellia oleifera* fruit example images that were generated by the Grad-CAM method (Figure 8), the model was well-trained because it identifies similar regions within the same maturity stages and different regions across the maturity stages, which may improve the accuracy of the *Camellia oleifera* maturity identification model. Interestingly, the regions of focus of the model were similar within the maturity of *Camellia oleifera* but different across maturities. For instance, for unripe *Camellia oleifera*, the model focused on the peel regions (Figure 8a,b). However, for ripe *Camellia oleifera*, the model focused on the crack regions and no longer on the peel regions (Figure 8c,d). Moreover, for overripe *Camellia oleifera*, the model focuses more attention on the camellia seed regions (Figure 8e,f). These behaviors are seemingly reminiscent of how human experts would estimate *Camellia oleifera* maturity, either focusing on the crack and camellia seeds regions if the peel is dehiscent or observing the peel regions when the *Camellia oleifera* shows no dehiscent.

**Figure 9:**
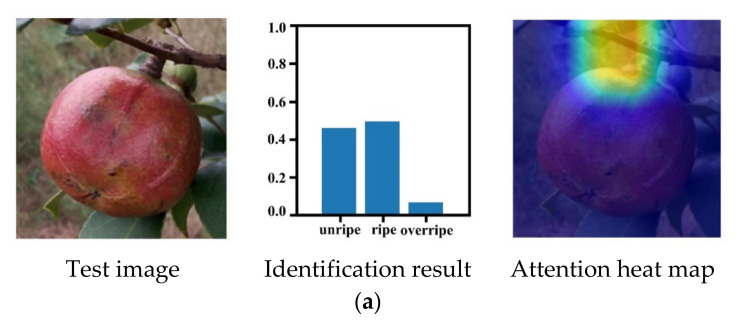

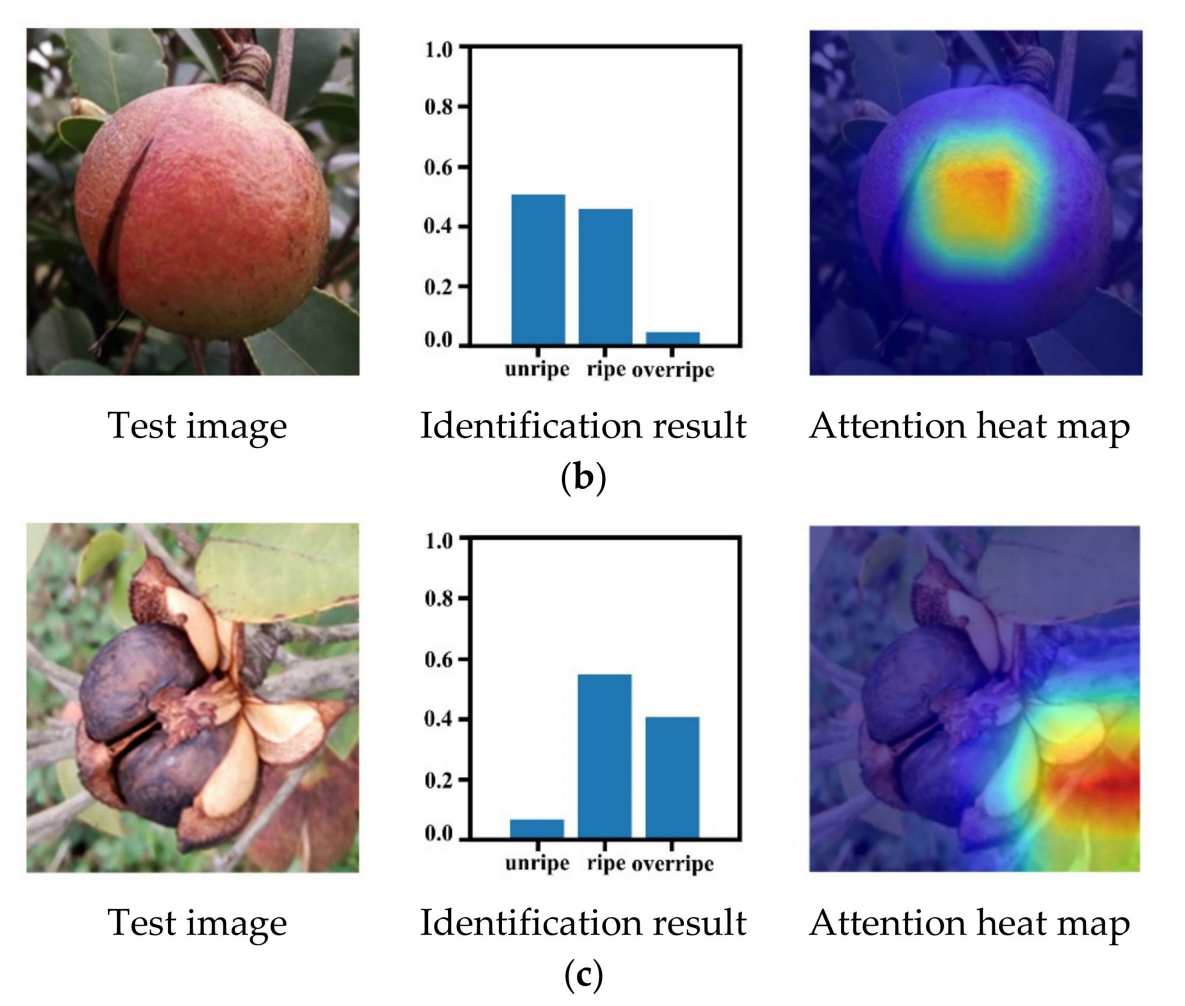
Some example heat maps which identified the maturity stages of *Camellia oleifera* by the proposed model incorrectly: (**a**) unripe identified as ripe, (**b**) ripe identified as unripe, and (**c**) overripe identified as ripe.

However, our maturity identification model also misidentified the maturity of some *Camellia oleifera* fruits. Figure 9 presents some example heat maps that misidentified the maturity stages of *Camellia oleifera*. The Grad-CAM analysis revealed that the incorrect identification of *Camellia oleifera* maturity was caused by failing to locate the typical region of focus for maturity identification. For example, when identifying unripe *Camellia oleifera*, the model incorrectly focused on the non-fruit regions, which resulted in unripe *Camellia oleifera* being mistakenly identified as ripe (Figure 9a). Besides, the model failed to accurately focus on the crack region when identifying the ripe *Camellia oleifera*, resulting in ripe *Camellia oleifera* mistakenly identified as unripe (Figure 9b). Thus, training with a higher number of *Camellia oleifera* images and adopting an attention mechanism may be another suitable strategy to improve the effectiveness of *Camellia oleifera* maturity identification [66]. In addition, the *Camellia oleifera* in the background also influences the ultimate results of *Camellia oleifera* maturity identification (Figure 9c). Therefore, although the model may potentially be capable of identifying *Camellia oleifera* maturities, further model improvements will be needed for practical applications to adapt to the complex environment.

## 4. Conclusions

In conclusion, to acquire high-quality and high-yield Camellia oil, it is indispensable to grade and identification the maturity of *Camellia oleifera* fruits. This study developed a *Camellia oleifera* fruit maturity grading and identification method based on unsupervised image clustering techniques. Measurements of overall accuracy indicated that the proposed method successfully achieved 91.25%. Moreover, Grad-CAM visualization analysis suggests that peel regions, crack regions, and seed regions were the key positions for *Camellia oleifera* fruit maturity identification. Besides, the *Camellia oleifera* fruits with ripe maturity stages were the most suitable for harvesting. The misidentification occurred due to the phenotypic properties of adjacent maturity *Camellia oleifera* visually appearing more similar to each other. In addition, the complex environment also had an impact on the maturity identification of *Camellia oleifera* fruits. The limitations of this study will be changed by acquiring more *Camellia oleifera* fruit images and suppressing the complex environment. Moreover, the proposed *Camellia oleifera* fruit maturity grading and identification methods are still wide open for further enhancement to simultaneously identify the maturity of multiple *Camellia oleifera* fruits in the image. The current findings contribute towards refining the harvesting process of *Camellia oleifera* fruits and facilitate the *Camellia oleifera* industry to improve quality and efficiency.

## Figures and Tables

**Table 1:** Dataset overview.

Dataset Category	*Camellia oleifera* Fruit Images	*Camellia oleifera* Fruit Samples
TN	2628	160
Unripe_TS	50	50
Ripe_TS	60	60
Overripe_TS	50	50

**Table 2:** Physical properties of *Camellia oleifera* with different maturity stages.

Physical Property	Maturity Stage		
	Unripe	Ripe	Overripe
Transverse diameter (mm)	46.05 ± 1.54 a	47.31 ± 1.65 a	49.32 ± 1.57 a
Vertical diameter (mm)	38.78 ± 1.33 a	40.10 ± 1.95 a	40.64 ± 1.71 a
Dry seed weight (g)	8.06 ± 0.41 c	12.44 ± 1.02 a	11.05 ± 0.38 b
Moisture content (%)	69.55 ± 3.74 a	58.79 ± 2.13 b	48.15 ± 0.62 c

Values are the mean of three independent determinations ± standard deviation. Different letters in the same row indicate significant differences (*p* < 0.05).

**Table 3:** Quality properties of *Camellia oleifera* with different maturity stages.

Quality Property	Maturity Stage		
	Unripe	Ripe	Overripe
Seed oil content (%)	39.12 ± 0.99 c	46.05 ± 0.49 a	44.42 ± 0.53 b
Seed-soluble protein content (%)	5.21 ± 0.22 c	5.61 ± 0.16 b	6.11 ± 0.07 a
Seed-soluble sugar content (%)	25.69 ± 1.16 a	19.28 ± 3.85 b	13.75 ± 0.93 c
Seed starch content (%)	3.31 ± 0.55 a	2.30 ± 0.24 b	1.54 ± 0.82 c

Values are the mean of three independent determinations ± standard deviation. Different letters in the same row indicate significant differences (*p* < 0.05).

**Table 4:** Quantitative evaluation results of the identification of *Camellia oleifera* fruit maturity.

Maturity	Evaluation Index			
	*Prec* (%)	*Rec* (%)	*F*1*_score_* (%)	*OA_cc_* (%)
Unripe	94.12	96.00	95.05	91.25
Ripe	89.66	86.67	88.14	
Overripe	90.20	92.00	91.09	

*OA_cc_* (%) represents the overall maturity identification accuracy for all three maturity stages.

## Data Availability

The datasets used and analyzed during the current study are available from the corresponding author on reasonable request.
